# Limited open information sharing and mobility promotes sustainability of jaguar tourism in Pantanal wetland, Brazil

**DOI:** 10.1038/s41598-024-72906-x

**Published:** 2024-10-01

**Authors:** Fernando Tortato, Alice Gottesman, Rafael Hoogesteijn, Abigail Martin, Mark Dyble, Rafael Chiaravalloti

**Affiliations:** 1 Panthera, New York, NY USA; 2 Department of Anthropology, University College of London, London, UK; 3 Jaguar ID Project, Poconé, Brazil; 4 Department of Archaeology, University of Cambridge, Cambridge, UK; 5 Department of Ecology, Lab of Movement and Population Ecology, Federal University of Mato Grosso do Sul, Campo Grande, Brazil

**Keywords:** Common-pool resource, Pantanal, *Panthera onca*, Property theory, Wildlife tourism, Conservation biology, Environmental economics, Sustainability

## Abstract

Wildlife tourism plays a crucial role in biodiversity conservation. However, long-term sustainability is difficult to achieve. In this paper, we use property theory to produce a mathematical model that aims to better support stakeholders from the wildlife tourism industry to better guarantee a balance between sightings probability, tourists’ overall experience and operators’ sharing behaviour. We illustrate our model with the case study of Porto Jofre in the Pantanal wetland, Brazil. We show that while dealing with low sighting probability, tourist operators must share information about species’ locations, leading to a system of open access regarding mobility and information. However, when sightings become common, sharing must be restricted to a bounded group avoiding overcrowding, a system of limited open access. Finally, when the sighting probability is high, no sharing is needed to achieve maximum overall experience. Our case study in Porto Jofre, Pantanal, Brazil, clearly shows these shifts in terms of governance strategies. We show that by looking at sighting probability it is possible to predict the best optimal social strategy that will guarantee long-term sustainability of the wildlife tourism initiatives. We also show the need for external support on adaptation in cases where current strategies do not match the predicted ones.

## Introduction

Wildlife tourism plays a crucial role in biodiversity conservation^1^. It has contributed $120.1 billion to the global economy, surpassing the illegal wildlife trade’s value fivefold, and supported 6.8% of jobs in the travel and tourism sector^2^. By creating economic incentives for wildlife conservation, ecotourism becomes a pivotal tool in slowing the global extinction rate and preserving ecosystem services^3,4^. However, wildlife tourism, like most conservation initiatives, must find a balance between economic sustainability, public interest and biodiversity conservation to be successful^5,6^. Understanding these drivers is critical for promoting the sustainability of ecotourism and, ultimately, protecting biodiversity.

Some authors have used property theory to better understand and point out pathways to the long-term sustainability of wildlife tourism^7–9^. In a simplistic model, wildlife sightings can be understood as a common-pool resource that diminishes with many people using it (known as subtractability of the resource) and, in principle, everyone who wants to access it (known as commons in property theory) has the right to do it^7,8^. Consequently, wildlife sightings are under the threat of overpopulation of commons diminishing the resource to the point of collapse, known as the tragedy of the commons. For example, overcrowded places may reduce each user’s experience to a point that the initiative is no longer viable to tourists, or which may impact biodiversity in a way that the resource disappears. Property theory argues that the collapse can be avoided through clear limits on access such as limits on tourist numbers or restricting the areas that tourists can visit^9,10^. This can be done through privatization or state control (the owner controls the use) or by agreements among the different users in the area (a common property regime).

Many of the wildlife tourism schemes, however, fail to be resolved through the principles of property. According to the property theory, commons accessing resources must understand the possible pay-offs of limits to accept them. However, wildlife sightings that rely on naturally inconspicuous species or which, normally, are afraid of humans due to a history of conflict, are under high levels of uncertainty around the possible pay-off. Therefore, the property theory solution focused on restricting the areas that tourists can access to avoid the tragedy of the commons may lead to low pay-offs, and tourists may stop coming to see something that may not happen.

As a solution for the challenge of sustainability in unpredictable systems, researchers argue that socio-ecological systems tend to have a rather opposite structure predicted by the property theory when access is uncertain^11^. They argue that sustainability “a balance between a “good” pay-off and level of use that does not impact the long term existence of the resource” is reached through fuzzy boundaries allowing people to move throughout the landscape and high levels of sharing around either where to find resources or the resource itself. In other words, rules focused on increasing the chance of accessing the resources instead of clear limits between people and the resources they use^12^. Therefore, depending on the level of predictability, users could display no cooperation, fully cooperation where everyone share information and limited cooperation where information is shared with a restricted number of people. However, the theory of sustainability under uncertainty has never been tested and evaluated for wildlife tourism.

To explore these questions, we present the case study of jaguar (*Panthera onca*) tourism in Porto Jofre, Pantanal, Brazil, and explore it through the new theory on property under unpredictability conditions. Based on secondary information, semi-structure interviews, long-term ethnographic data of the region, and a survey, we present how tourist operators built different strategies of mobility and information sharing depending on levels of sighting’s success. We also produced a mathematical model that tested the best sharing strategies in order to deal with different levels of environmental predictability of wildlife sightings. Our goal is to explore the long-term sustainability of wildlife observation in places where resources are unpredictable and the implementation of clear limits are unfeasible. Ultimately, we aim to support scientists, practitioners and other players of the wildlife tourism industry struggling to reach sustainability under unpredictable dynamics.

## Methods

### Study area

**Fig. 1:**
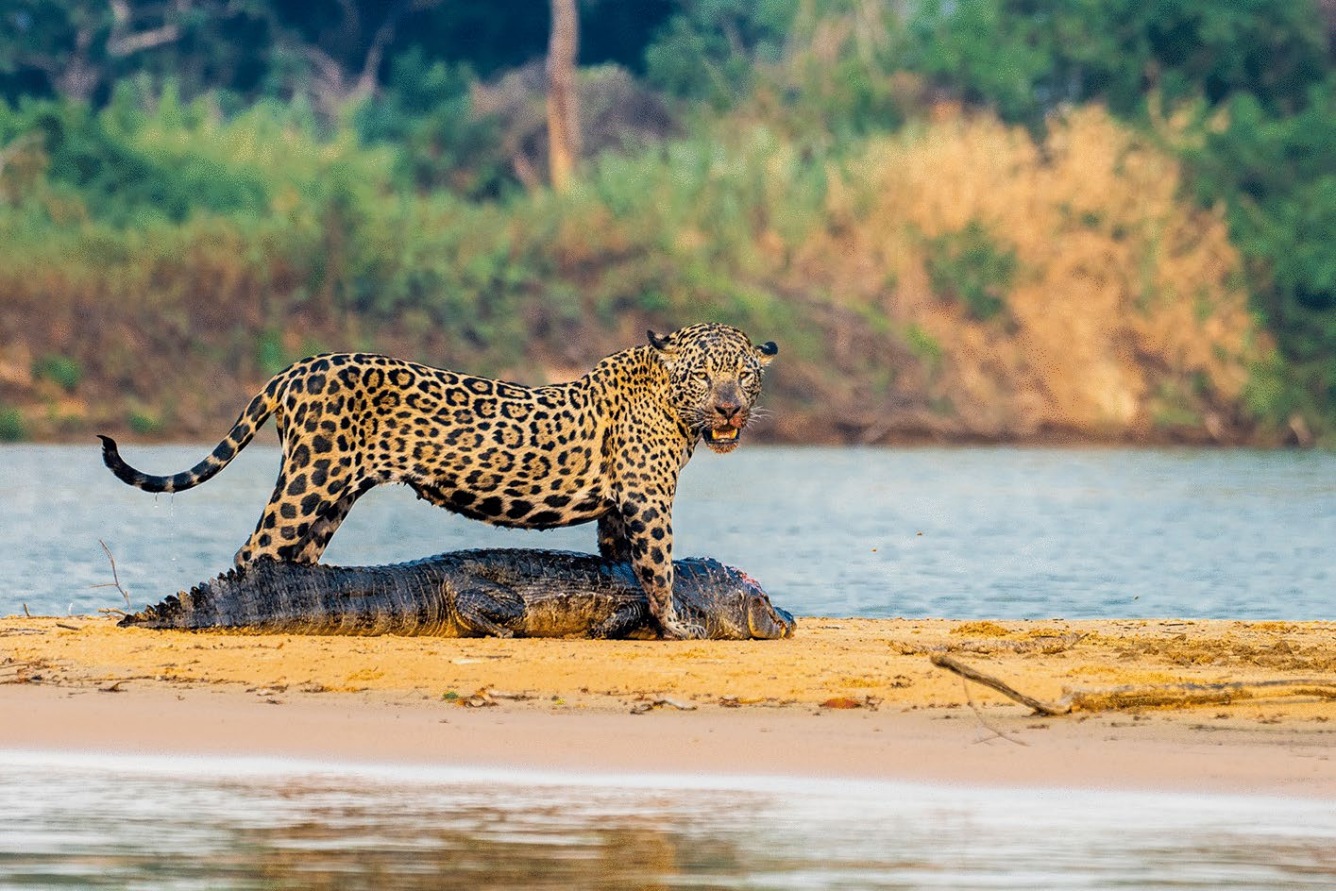
A jaguar (*Panthera onca*) with its prey, a caiman (*Caiman yacare*), being watched by tourists in the Pantanal (Credits Sebastian Kennerknecht).

Porto Jofre is located in the 179,300 Km^2^ Pantanal wetland (Fig. 1)^13^. In the 1960s and 1980s^14^, the Porto Jofre region was particularly impacted by poaching and the jaguar had virtually disappeared from the region. However, from 2000s onwards, wildlife tourism, especially focused on jaguars, allowed the region to undergo a remarkable transformation into a thriving hub for wildlife observation despite the species’ inconspicuousness and history of conflict with humans^15^. Today the Pantanal contains the world’s second-largest jaguar population and one of the highest recorded jaguar density^16,17^, ranging from 4.08^18^ to 12.4 animals per 100 km^2^^16^. Presently, jaguar tourism generates a gross annual income of US$6,827,392 in land-use revenue, reflecting the transformative impact of the operation^19,20^.

## Data collection and analysis

### Semi-structured interviews and participatory mapping

To capture the governance structure and history of ecotourism in the Porto Jofre region we relied on long-term ethnography of the region, semi-structured interviews, and secondary data. The ethnographic observations are based on monthly visits to the area for the last 15 years by one of the authors (FRT). This knowledge helped us to understand the socio-ecological system and support the development of the semi-structure interviews. It also helped to identify key informants who would have long-term experience in the area. We used a set of open-ended questions, which allowed interviewees to explore the topic in more depth (supplementary material). All interviews were recorded and transcribed. We analyzed the interviews by examining the answers for common patterns to identify relevant themes, and then information was extracted for each theme and synthesized. The purpose of the analysis was to create a storyline linking the different aspects together into a coherent account^21^. In total, we interview 44 local people. Given our long-term experience in the area, we estimate that this accounts for 30% of all people involved in the jaguar tourism in the region. We also conducted participatory mapping with 12 people of those which we conducted the semi-structure interviews. The method consisted of printing satellite images of the area in vinyl papers and asking people to draw the areas they normally take tourists. Our goal was to spatialize their knowledge of the jaguar tourism operation and allow for more meaningful conversations about how jaguar sighting hotspots are governed^22^. Finally, as noted above, one of the co-authors (FRT) has over 15 years’ experience in the area working with tourist operators: this ethnographic knowledge was incorporated into the description of the governance structure and history of jaguar tourism operations.

### Mathematical model

The mathematical model to elucidate the governance mechanisms of wildlife sighting under different levels of predictability was drawn from Chiaravalloti and Dyble’s (2019). The parameters were adapted according to the experience given by the case study in Porto Jofre, Pantanal.

### Parameters of the model in Porto Jofre, Pantanal

To test the wildlife observation model for the case study of Porto Jofre we also carried out a survey with tourist operators working in the region. We sought to capture the two main parameters of the model—(a) number of boats in the river and (b) the predictability to sight a jaguar. For Porto Jofre, experts suggested dividing the parameters for the low and high season. To find an accurate answer for these questions, we employed a structured expert protocol named IDEA (“Investigate,” “Discuss,” “Estimate” and “Aggregate”). The protocol aims to systematically quantify experts’ knowledge on information about patterns that are complex to address. The protocol is based on two steps. First, experts in the topic are asked to give their best estimate, confidence interval, and the degree to which the responses accurately captured the true value. Following an initial round of individual interviews, all experts’ answers are shared and the group debate and revise their personal answers. Ultimately we aim to gather a collective response for the questions. Our group of experts were composed of 11 people. We chose the most experienced tourist operators in the area. We carried out 11 interviews—five local boat drivers and six tour guides. We focused on two key questions during the interviews: (1) the number of tourist boats observing the same jaguar, and (2) the probability of a tourist encountering a jaguar within 10 days.

### Ethical approval

We confirm that all methods were carried out in accordance with relevant guidelines and regulations. All research activities and processes were approved by the University College London (UCL) Anthropology Ethics Committee (approval number: UCL-ANTH/PGT/22–23/016). We confirm that we had the informed consent of all participants and/or their legal guardian(s) for data collection. Before starting the interviews, we explained who we were, what the research study was about and how the resulting data would be used. We then confirmed whether the research participant wanted to proceed with the applicable research exercise(s). Participants were informed that the data used was for the exclusive purpose of the research and that the personal data obtained would be kept confidential.

## Results

### Governance structure enabling jaguar sightings in Porto Jofre

Tourist operators in Porto Jofre take dozens of tourists on daily boat trips to sight jaguars. Porto Jofre is located in a large floodplain area of the Cuiabá River, with many tributaries that facilitate access hotspots for jaguar. Tourist operators freely move throughout the floodplain in search for jaguars. There are no limitations on where boat drivers and tour guides can take tourists. A boat searching for jaguars will often travel dozens of kilometres across the rivers each day until it finds a jaguar. Many times, they trespass both state and private properties. For instance, even though there a strictly protected area in the region, which in principle would require tourist operators to grant access from government agencies, no enforcement is imposed. As reflected by two boat drivers who commented, “*There are no limits. It just depends on how easy it is for us to do the sighting. If we go somewhere and cannot find them, we will go elsewhere*” (Int.1).

The only limits are related to their contact with jaguars, which is regulated by both local agreements and government resolutions. These resolutions are considered pioneering in Brazil’s wildlife tourism industry because they set rules such as maximum observation time, minimum distances between the boats and jaguars, and the number of boats allowed in an area^15^. Some of the local agreements are more restrictive than the government ones. For instance, according to the Mato Grosso state (the state where Porto Jofre is located), the minimum distance is 10 m (local resolution number 86/11), but the Civil Association of Ecotourism of the North Pantanal established a distance of 25 m. However, the effectiveness of self-regulation is constantly challenged. Sometimes is possible to see overcrowded areas with distance between boats and wildlife of less than a meter.

To maximize the success of the search for jaguars, guides used to have an open-frequency radio communication—the parallel of open access in property theory. Thus, if a tourist operator would find a jaguar, s/he would communicate in the radio the location of the jaguar to all other tourist operators, who will take the tourists to that specific location. By sharing information about the location of jaguars, they were aiming to increase everyone’s likelihood of jaguar sightings. It was a way to guarantee that tourists are able to observe a conspicuous species during a normally short visit in the region. Given the confidence with the certainty to observe a jaguar in the region, today tourist guides and companies offer a “guaranteed” package to tourists in the Porto Jofre Region. Some tour operators have already offered a full reimbursement if tourists do not see a jaguar during a three-day package in the region.

Recently, however, a citizen science Jaguar ID Project has shown that more jaguars in the region started to get habituated with the human presence. According to the programme which records jaguars from photos taken by tourists/tourist operators and identify them the number of jaguars habituated went from 29 in 2013 to 130 in 2023, an increase of over 400% in ten years (https://www.jaguaridproject.com/). The increased ease of detecting jaguars coupled with the rising number of boats on the rivers and open frequency radios started to lead to overcrowding. Some places started to see dozens of tourists all at once sighting one jaguar. Many challenges started to appear. Our ethnographic and semi-structure interviews in the region shows that there is a perception of possible increase of poor experience for the tourists, risks of boats encroaching on jaguars, limitation to jaguar mobility (e.g. swimming) and hunting activities, which risks the economic sustainability of the business model and also the species conservation.

Our ethnographic and semi-structure interviews have also shown that in order to deal with overcrowding, some tourist operators stopped openly sharing information in the radio and restricting it to a small number of people (known in property theory as limited open access), forming bounded groups. Interviews with tourist operators have shown that the bounded system is normally related to the hotel lodge that a specific tourist operator works for, however, there are also information sharing based on friendship and affinity. In some cases, tourist operators said they no longer share information at all.

### Mathematical model to characterise governance of wildlife observation

We first assume that wildlife tour operators ultimately aim to achieve the best possible experience (*E*) of jaguar sighting for tourists. The model explores how tour operators would achieve this under varying degrees of predictability in wildlife sighting. We argue that different levels of predictability may require different levels of information sharing between tour operators. We consider three possible options available to tour operators. If someone finds a jaguar the information about its location will: (1) not be shared—a ‘no cooperation’ system, (2) shared with a limited number of people—a ‘limited open access’ system, (3) shared with all tourist operators that are in the river—an ‘open access’ system.

It is important to state that we do not argue that tour operators do not have other interests other than tourists’ experience. For instance, personal decisions, lack of background on tourism initiatives, and lack of commitment with the local sustainability all may affect tourist operators’ decisions. However, for simplicity and generalization of the model we adopted this approach.

We model the experience of jaguar sighting (*E*) as the probability of a sighting (*P*) multiplied by the quality of that sighting (*Q*): *E* = *QP*. The quality of the sighting can vary between 0 and 1 where 1 is an ideal sighting in which the tourists observe a jaguar without any other tourist boats in the vicinity. A sighting of *Q* = 0 is considered as so poor that it is equivalent to not observing the jaguar at all. The justification for multiplying *Q* and *P* is that this is equivalent to averaging across two binary possibilities: sighting the jaguar (with probability *P*) and having a sighting of quality *Q* ranging between 0 and 1 or else not observing a jaguar (with probability 1 – *P* and, in effect, *Q* = 0). For example, if a tourist have an 80% chance (*P* = 0.8) of seeing a jaguar, with a sighting quality of *Q* = 1 then the expected experience ahead of the tour is 0.8 × 1 = 0.8: there is an 80% chance of *Q* = 1 and a 20% chance of *Q* = 0. Our goal is to model the conflict that tourist operators face: on one hand a system of information sharing about the location of jaguars will increase their chancing of a sighting (*P*) but may lead to a crowd of boats that diminish the quality of the sighting (*Q*) for tourists. Of course, other factors may also play a role in the experience of jaguar sighting such as weather conditions, quality of the lodge, tourists’ personal interest, age groups, and so on^23,24^. However, for simplicity and generalization we adopted this approach.

### Probability of a jaguar sighting (*P*)

Probability of a jaguar sighting (*P*) is influenced by both biological and social factors. Biological factors encompass jaguar’s density in the area, proximity to the river, habituation, season, weather, among others. We consider all of these factors to contribute to a model parameter *b* that represents the baseline chance of a single boat locating a jaguar in a single day without assistance. Social factors for the area are mainly related to numbers of tour operators that are sharing information about jaguar locations. The number of tour operators is represented in the model by the parameter *g.* For simplicity and generalization, we consider that all guides have the same level of prior information and knowledge about the area: we do not account for the fact that the probability of a sighting may vary between tour operators as a function of their experience.

**Fig. 2:**
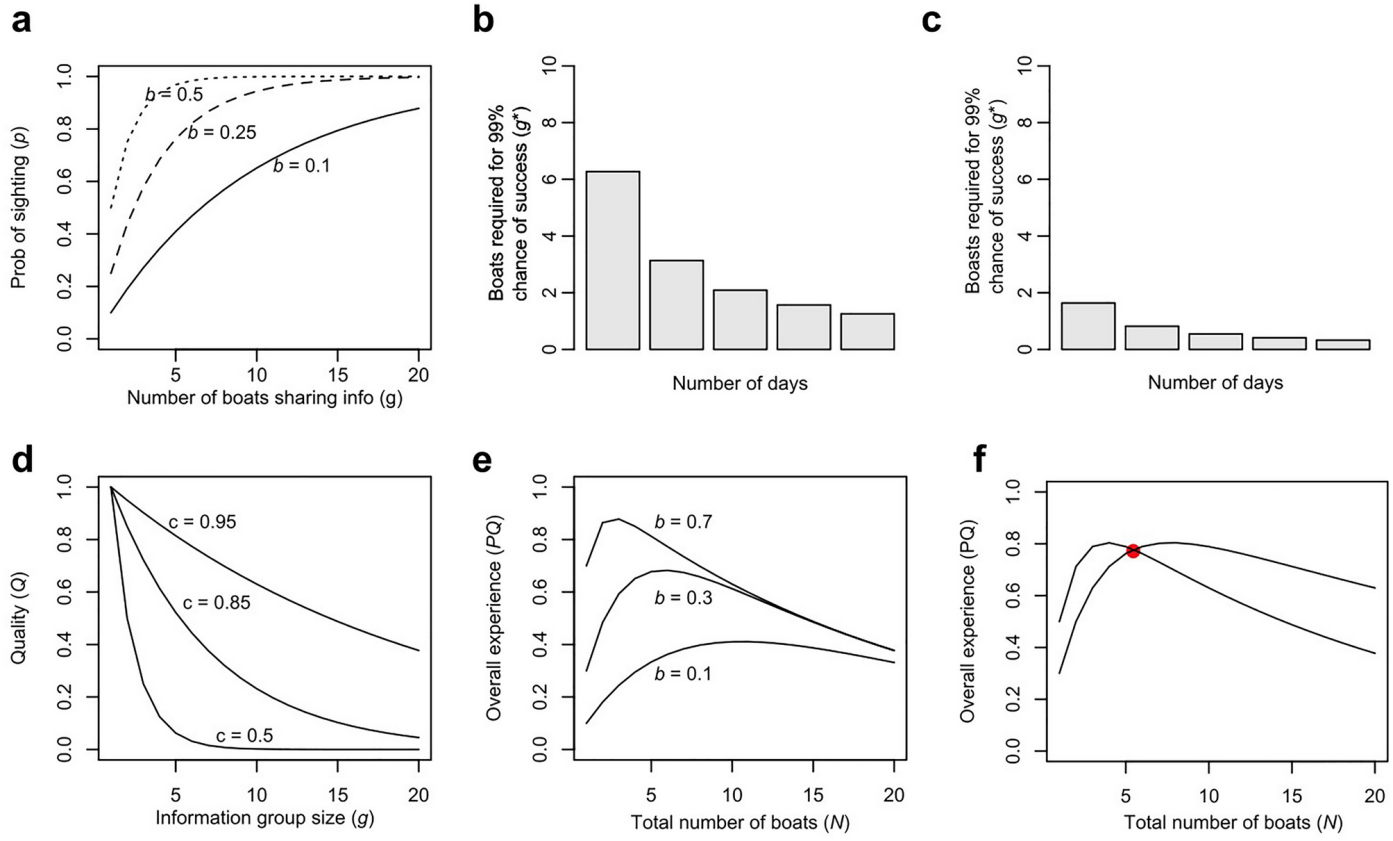
(**a**) Predicted probability of a sighting (*P*) at *b* = 0.1 (solid),* b* = 0.25 (dashed) and *b* = 0.5 (dotted) (**b-c**) Number of boats required for a 99% probability of jaguar sightings, considering durations ranging from 1 to 5 days on the river, with a baseline probability set at the low season baseline (b = 0.52) in a and the high season baseline (b = 0.94) in b. (**d**) Variation in quality relative to group size, depicting the influence of distinct levels of competition among boats. (**e**) Overall expected tour experience in relation to the size of the information sharing group (*g*). (**f**) The expected overall experience comparing open information sharing among all boats (*g* = *N*) and cooperative open access with two separate information-sharing groups (*g* = *N*/2). The red dot denotes the crossover point where being in a group of *N*/2 boats becomes preferable to a group of* N* boats (in this instance, occurring when *g* = 9).

Looking at the ecological drivers, we can say that the probability of a single boat *not* locating a jaguar in a single day is: 1 – *b*. Considering that we have several boats looking for jaguars in a single day (*g)*, we can say that the probability that none of the boats in the group locate a jaguar is: (1 – *b*)^*g*^*.* This allows us to conclude that the probability that at least one group member makes a sighting in a single day is: 1 – (1 – *b*)^*g*^. We assume that any boat making a sighting in the sharing unit will share this information and that all other boats will also succeed in seeing a jaguar at that location. Figure 2a shows *P* across values of *b*.

We also must consider that tourists spend at least a few days in the region to enhance the likelihood of a successful jaguar sighting, here considered as *d*. Therefore, we can multiply the chance of seeing a jaguar by the number of days the tourists would stay in the region, such that the probability of a sighting given *g* tour operators searching over *d* days is 1 – (1 – *b*)^gd^. These probabilities are different in low season (Fig. 2b) and high season (Fig. 2c).

To understand the level of sharing that would give a certain probability a jaguar sighing according to different levels of ecological unpredictability, we can rearrange the expression 1 – (1 – *b*)^gd^, solving for *g*, to give the size of information-sharing group (*g*) required for a tour guide to offer a specific probability of a sighting (*s*) over a specified number of days (*d*). This required group size is denoted as g*.

1 $${g}^{*}= \frac{log\sqrt[d]{1-s}}{log(1-b)}$$

For instance, if the probability of sighting a jaguar is *b* = 0.3 and the tourist operators aim to provide tourists a package of three days in each they will “guarantee” a sighting (operationalised as a chance of at least 99%) then g* would be 4.30; signifying that a minimum of five boats actively sharing information is necessary to achieve this 99% probability of seeing a jaguar in three days.

### Quality of the jaguar sighting

Another important aspect of a tourist’s experience is the quality of the sighting. For simplicity of the model, we consider that quality is only influenced by the number of tourists seeing the same animal. Ultimately the maximum quality (Q = 1) is reached when a tour operator takes the tourist to observe the jaguar with no other tourists around. However, in reality, quality is driven by uncountable aspects, ranging from animal behaviour during the observation to weather and tourists’ own feelings.

In this model, we express quality (*Q*) as *c*^(*g* − 1)^. Here, *c* represents a parameter influencing subtractability: the degree to which the quality of the sighting experience diminishes when shared with other boats. Figure 2d illustrates the impact of *c* on *Q* across various information-sharing group sizes.

### Overall experience and governance strategies

Given that we have expressions for quality and probability of a sighting, we can estimate what are the best sharing strategies to achieve maximum tourist experience under different levels of sighting predictability. When plotting the overall experience against information-sharing group size, we observe that, for certain combinations of *c*, *g*, and *b*, there exists an optimal group size, striking a balance between the probability and the quality of the sighting. Figure 2e illustrates this scenario for *b* = 0.3, *c* = 0.95, and varying values of *g*.

If the total number of boats on the river is *N*, then the expected point at which experience would be improved by splitting into two groups is where the overall experience (*E*) is greater at* g* = *N*/2 than at *g* = *N* (Fig. 2e). In Fig. 2f, we illustrate the point at which this transition occurs, considering *b* = 0.3, *c* = 0.95, and varying values of N.

Our model also suggests what may happen to tourists’ experience when operators do not seek the optimal strategy in terms of sharing. For instance, when the chance of sighting a jaguar is *b* = 0.2, the subtractability is *c* = 0.90, in a package of *d* = *3* days*,* and they share information in group of *g* = 3 tour operators, tourists would have both a very good chance of a sighting (*P* = 0.96), a good quality (*Q* = 0.81) and an overall expected experience of *E* = 0.78. Given the same situation in terms of number of days, subtractability and the chance of sighting a jaguar, if tourist operators share information with, say, 10 boats (*g* = 10), the probability of a sighting would be only slightly higher (P ≈ 1) but the quality of the sighting and overall experience would be much diminished (E = Q = 0.39): they would have a high probability of seeing a jaguar but likely in the presence of many other boats.

### The model and the Porto Jofre jaguar tourism

The model helps to explain some of the patterns we see in the Porto Jofre jaguar tourism program. Initially, the number of jaguars that tourists were able to sight was low due to the history of conflict in the area (e.g. 29 jaguars habituated in 2013). As a consequence, the governance structure was based on sharing information with all boats/tourist operators that were in the river (open access, N = *g*). However, as more jaguars were habituated (high *b,* 130 habituated jaguars in 2023), sightings started to become overcrowded. Thus, many of the tourist operators started to close their radio frequencies to only fewer people, resulting in a system of limited open access (g < N). Our survey shows that, currently, people estimated that in the low season, their chance of seeing a jaguar in a three day package with no information sharing is *b* = 0.52, and they would see an average of N = 4.45 boats/tourist operators on the river. Therefore, they would achieve maximum tourists’ overall experience by sharing information with three people. This is essentially what we see on the ground, a limited open access system for the high season. However, we also saw that in the high season, they estimated that their chance of sighting a jaguar is *b* = 0.94% and they expect an average of 30 boats on the river. In this case, our model predicts that they would require no cooperation to maximize the tourist experience. However, on the ground, our qualitative research showed that they still share information with a small number of boats, potentially reducing the tourists’ expected experience.

## Discussion

Wildlife tourism is an important tool to slow down the current global species extinction rate and loss of ecosystem services^3^, and to promote local communities’ well-being^25^; however, creating sustainable wildlife sighting schemes where access to species is unpredictable is a challenge for tour operators all over the world^26^. In fact, many operators offer a refund if the tourist does not see the desired species, to keep the activity attractive^27^. Here, through a recently extended version of property theory, we present a mathematical model illustrated by a real case study on how to create a wildlife sighting scheme under unpredictable resources. This is critical, especially for charismatic species such as big cats, in which although they bring many benefits for their conservation^28–30^ their elusive behaviour, and sometimes livestock predatory habits make it challenging to implement^19^.

**Fig. 3:**
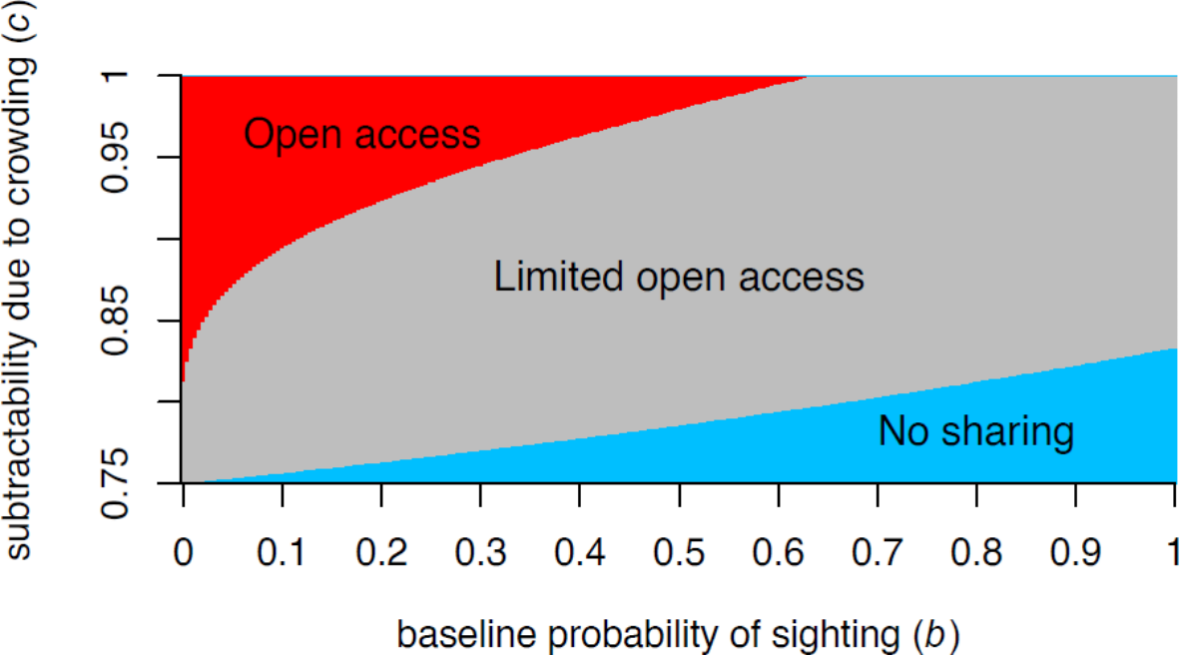
Information sharing system that would maximise tourist experience (E) given varying levels of subtractability due to crowing (c) and baseline probability of a sighting (b) in a situation with 8 tour operators on the river. Note that lower values of c indicate a stronger crowding effect: lower c means that the experience of tourists diminishes more rapidly for every additional tour they share a sighting with. Blue = conditions favouring no sharing, red = conditions favouring free information sharing with all operators (open access), grey = conditions favouring information sharing within small groups (limited open access).

We show that through mobility and different levels of information sharing, users can organize themselves to increase the chance of accessing resources and delivering high satisfaction for tourists while avoiding overcrowding. In summary, we show that while chances of sighting a wildlife are low, tourist operators should openly share information about the location of the animal. For instance, for jaguars, although sighting is only possible in less than 5% of its current distribution^31^, the local people in Porto Jofre managed to implement a successful case study. However, soon the probability of sighting the animals becomes higher (due to habituation, population increase, etc.), operators should move to a bounded system where sharing information is limited to fewer people, what we called a limited open access system. Finally, at higher chances of sighting the animal, no information should be shared to avoid overcrowding and guarantee tourist’s overall experience. Our paper presents a clear guidance on how to find this balance using simple parameters such as probability of sighting the animal, number of days tourists stay in the region and number of tourist operators. Although there are other variables that could be added in the model (such as tourist operators’ experience, weather, season), the model could be replicated in, virtually, any situation (Fig. 3).

Our model also helps to explain the failure to achieve long-term sustainability in many wildlife tourism schemes. For instance, in Porto Jofre, during the high season sightings are extremely reliable (b = 0.98), however, most tourist operators still share information in a limited open access fashion. Our model shows that this is not the best strategy to guarantee overall tourists’ experience in the area, and in the long term, it may lead to negative results for the species as well as for people. Therefore, first, Porto Jofre system urgently needs management measures to prevent it from collapsing due to overcrowding, like so many other wildlife tourism destinations^32,33^. Our mathematical model may also help other systems to evaluate whether the strategies on the ground are indeed the best strategies for long-term sustainability. In summary, conservation initiatives should help local groups to adapt to the ecological dynamics by allowing mobility and celebrating information sharing while dealing with rare and conspicuous species (limited and open access), and limiting access when resources are more predictable (no cooperation in terms of information sharing).

It is important to also understand why many tourist operators, like in Porto Jofre, do not follow the optimal strategy that guarantees long-term sustainability. Decisions around governance structures (such as sharing information or not) should be understood as part of a complex multi-stakeholder landscape. In fact, the challenge to balance individual benefits with collective sustainability (the collective action dilemma) is considered one of the greatest challenges for socio-ecological systems^34^. For resource-dependent small-scale communities, this is normally facilitated by cultural and family ties where people have been negotiating the governance rules for hundreds or thousands of years. On the other hand, in regions occupied by private companies, protected areas, ranchers the negotiation around governance rules are more difficult to acchieve^35^. Also, small-scale societies tend to have a low impact on natural resources regardless of the governance structure, which normally allows them to have a long history of wrong choices in terms of resource use before overcoming the collective-action dilemma^34,36^. Whereas, in most large, multi-stakeholder landscapes there is not much room for trial and error, given the tremendous threats these systems already face from precedent negative consequences of global changes for biodiversity, sustainability, and global health^37^. For wildlife tourism, for instance, few years of overcrowding can quickly collapse the area. Therefore, although our model presents a clear indicative of optimal types of information sharing depending on levels of predictability, adaptation has to be scaffolded by external and local partners. This may include (i) the identification of those areas where overcrowding or conflicts among competing ecosystem services may be pronounced (e.g. hotspots of trade-offs); (ii) making the need to long-term sustainability of ecotourism visible to decision makers and local stakeholders; (iii) emphasize the multiscale and multi-attribute nature of recreation, ecotourism and other land use, applying a territorial (landscape) rather than sectoral (local) approach for planning; (iv) complement integrated assessments of ecosystem services aimed at evaluating trade-offs under rapid landscape changes that have been documented in the study area (i.e. wildfires); (v) to orient investments and incentives on collective learning, education, and communication between tourist operators; (vi) to understand all positive and negative externalities of all activities in the landscape in a constant adaptative management^38^.

## Concluding remarks

The success of wildlife sightings is still not a resolved problem. In this paper, we present some initial insights into how people should best organize themselves in terms of information sharing and mobility to promote maximum tourist experience while avoiding overcrowding. We argue that in cases which sigh probability is very low, tourist operators should share information in a system with free mobility (open access system). This could be the case of most large carnivores or conspicuous species living in dense vegetation. While this probability starts to increase, tourist operators should change to a system where information and mobility is limited (limited open access). For instance, species that are rare yet are seen with a certain frequency. Finally, in areas with common sightings, no cooperation should be implemented—otherwise it would create a poor experience. By following this simple structure, we argue that wildlife tourism could be implemented for any species or ecosystem. It is also important to consider the adaptations needed according to changes on predictability, rather than a strict rules focused on controlling access and mobility. We also underscore the need for support on adaptation. Governance is a complex phenomenon especially in complex, multi-stakeholder landscapes. People may not adapt in the same speed of the changes on unpredictability of resources. Therefore, it is critical that government, NGOs, tourism entrepreneurs and guides, and the local communities constantly discuss and find common solutions that are able to, ultimately, guarantee of the best strategy for both biodiversity and people.

## Data Availability

The data that support the findings of this study are available from the corresponding author, FRT, upon reasonable request.
